# Projected Habitat Contraction and Distributional Shifts of the near Threatened Undulate Ray *Raja undulata* Under Climate Change

**DOI:** 10.3390/biology15131035

**Published:** 2026-06-29

**Authors:** Cemal Turan, Alen Soldo

**Affiliations:** 1 Department of Molecular Biology and Genetics, Faculty of Engineering and Natural Sciences, Atlas University, 34403 İstanbul, Türkiye; 2 Faculty of Marine Science, University of Split, 21000 Split, Croatia

**Keywords:** *Raja undulata*, species distribution modelling, MaxEnt, climate change, habitat suitability, conservation

## Abstract

Climate-driven changes in oceanographic conditions are increasingly affecting the distribution of marine species, particularly vulnerable elasmobranchs. The undulate ray, *Raja undulata*, is a Near Threatened batoid species distributed throughout the northeastern Atlantic Ocean and parts of the Mediterranean Sea, yet its potential response to future climate change remains poorly understood. This study assessed current and future habitat suitability using species distribution modelling approaches and CMIP6 climate projections under the SSP245 scenario. Species occurrence records were compiled from biodiversity databases and published sources, and environmental predictors were selected following multicollinearity screening. Among twelve evaluated modelling algorithms, MaxEnt showed the highest predictive performance (AUC = 0.99; TSS = 0.95) and was selected for subsequent analyses. Current habitat suitability was concentrated along the Iberian Peninsula, the Bay of Biscay, the English Channel, and parts of the western Mediterranean Sea. Future projections indicated substantial habitat contraction, with habitat loss (57.3%) greatly exceeding habitat gain (2.2%), resulting in a southward redistribution of suitable habitats. Minimum phytoplankton concentration, sea surface temperature, and silicate concentration were identified as the most influential environmental predictors. Areas predicted to remain suitable under both current and future conditions may represent important climate refugia for the species. Overall, the results indicate that *R. undulata* is highly vulnerable to future environmental change and highlight the need to incorporate climate-driven habitat shifts into conservation planning, fisheries management, and long-term monitoring strategies.

## Simple Summary

Climate change is altering ocean conditions worldwide, affecting the distribution and survival of many marine species. The undulate ray *Raja undulata* is a coastal skate species found in the northeastern Atlantic Ocean and parts of the Mediterranean Sea that is already threatened by fishing pressure and habitat degradation. Understanding how future environmental changes may affect this species is essential for effective conservation. In this study, we identified the environmental conditions associated with suitable habitat for the undulate ray and predicted how its distribution may change under future climate conditions. Our results indicate that future habitat losses are likely to greatly exceed habitat gains, leading to an overall contraction and southward redistribution of suitable habitats. Environmental conditions linked to ocean productivity and temperature were identified as the main factors influencing habitat suitability. Although some regions are predicted to remain suitable and may serve as future climate refuges, many currently suitable areas are projected to decline. These findings provide valuable information for conservation planning, fisheries management, and long-term monitoring efforts aimed at protecting the species.

## 1. Introduction

The acceleration of global climate change poses one of the most pressing challenges to marine biodiversity and ecosystem functioning worldwide [1]. Rising ocean temperatures, ocean acidification, declining oxygen levels, and changes in ocean productivity are altering the structure and dynamics of marine ecosystems, forcing species to adapt their phenology, physiology, and geographic distribution in order to remain within their environmental tolerance limits [2,3]. In most cases, these responses manifest as the expansion, contraction, or complex redistribution of habitats and can affect interspecies interactions, ecosystem stability, and fishery resources [4]. For this reason, understanding how environmental changes can affect species distribution is vital for predicting biodiversity loss, safeguarding ecosystem services, and developing effective conservation and management strategies.

Species Distribution Models (SDMs) have become indispensable tools for assessing species-environment relationships and predicting potential changes in distribution under future climate scenarios [5]. By combining species sighting records with environmental factors, species distribution models (SDMs) can identify patterns of habitat suitability and predict the future distribution of species under alternative environmental conditions [5,6]. In marine environments, where long-term ecological monitoring is often limited in both space and time, species distribution models provide a robust framework for assessing species sensitivity and contributing to evidence-based management decisions [7,8]. The Climate Model Intercomparison Project Phase 6 (CMIP6) climate projections, which were recently made available, further enhance our ability to assess future habitat dynamics through the use of updated climate scenarios and improved environmental datasets [9].

Elasmobranchs (sharks, rays, and chimaeras) are among the marine taxa most vulnerable to environmental change and anthropogenic pressures. Many species exhibit life-history characteristics such as slow growth, late maturity, low fecundity, and long generation times, which limit their capacity to recover from population declines [10,11,12]. In *Raja undulata* Lacépède, 1802, delayed sexual maturity and relatively low reproductive output are considered particularly important constraints on population recovery, as they reduce the species’ ability to compensate for increased mortality caused by fishing pressure and habitat degradation [13,14,15]. Furthermore, numerous elasmobranchs occupy relatively narrow ecological niches and depend on specific environmental conditions, making them particularly sensitive to climate-driven changes in temperature, oxygen availability, and ecosystem productivity [16,17]. Consequently, understanding how environmental change may affect their future distributions has become an important priority for marine conservation and fisheries management.

The undulate ray *Raja undulata* is a demersal batoid species inhabiting coastal and continental shelf environments characterized by sandy and muddy substrates in the northeastern Atlantic Ocean and parts of the Mediterranean Sea [13,15]. As a mesopredator within benthic ecosystems, the species contributes to trophic regulation and ecosystem functioning. However, *R. undulata* has experienced substantial population declines throughout parts of its range due to fishing pressure, bycatch mortality, habitat degradation, and other anthropogenic impacts [14,18]. Consequently, the species is currently classified as Near Threatened on the IUCN Red List [15], highlighting the need for improved understanding of the environmental factors influencing its distribution and persistence.

Despite its importance for conservation, knowledge regarding the environmental factors shaping the distribution of *R. undulata* remains limited. Although the species’ geographic distribution has been relatively well documented, previous studies have largely focused on occurrence records, fisheries assessments, and regional ecological observations [14,18,19,20], while the relative importance of oceanographic and climatic variables governing habitat suitability has received limited attention. Furthermore, no comprehensive species distribution modelling study has evaluated the potential effects of future climate change across the species’ entire Atlantic and Mediterranean range using updated CMIP6 climate projections. In addition, the relative performance of multiple modelling algorithms and the contribution of key environmental predictors to habitat suitability have not previously been assessed for this species. Addressing these knowledge gaps is essential for identifying vulnerable populations, anticipating future range shifts, and supporting adaptive conservation planning under changing environmental conditions.

Therefore, in this study, we applied species distribution modelling approach to assess the current and future habitat suitability of *R. undulata*. Specifically, our objectives were to: (1) identify the key environmental variables shaping the species’ ecological niche, (2) predict its current habitat suitability and distribution, and (3) project potential future distributional changes under a climate change scenario in order to evaluate its vulnerability to ongoing environmental change. By identifying the environmental drivers of habitat suitability and forecasting future range dynamics, this study provides a scientific basis for the conservation and management of *R. undulata* under future climate conditions.

## 2. Materials and Methods

### 2.1. Species Occurrence Data

Occurrence records for *Raja undulata* were compiled from multiple global biodiversity databases accessed through the spocc R package, including the Global Biodiversity Information Facility (GBIF), iNaturalist (iNat), EcoEngine, VertNet, BISON, ALA, iDigBio, and the Ocean Biodiversity Information System (OBIS). Additional records were obtained from published literature to improve spatial coverage across the species’ known distribution range [14,18,19,20,21]. To ensure data quality, records from different sources were merged and duplicate occurrences sharing identical geographic coordinates were removed. Spatial outliers and records with inaccurate or invalid coordinates, including terrestrial locations and points falling outside the known distribution range of the species, were also excluded. The study area was defined based on the documented Atlantic and Mediterranean distribution of *R. undulata* reported in the literature and the spatial extent of validated occurrence records retained after data cleaning. The resulting dataset consisted of georeferenced presence-only records and was used for all subsequent species distribution modelling analyses.

Because the analyses were based on presence-only occurrence records, each georeferenced point represents a documented species occurrence rather than a measure of local abundance or population density. Consequently, individual records shown on distribution maps should be interpreted as indicators of species presence within a given area and not as isolated observations. In several regions of the north-eastern Atlantic, where *R. undulata* is known to occur at relatively high local abundance, available georeferenced records may nevertheless be represented by a limited number of occurrence points due to uneven sampling effort, data availability, and reporting practices.

### 2.2. Environmental Predictors

A total of 21 environmental variables describing oceanographic and climatic conditions were initially considered for modelling habitat suitability. These variables represented key environmental gradients known to influence marine species distributions, including temperature, salinity, dissolved oxygen, nutrient availability, primary productivity, seawater density, pH, and suspended matter [7,22]. All environmental layers were standardized to a common spatial resolution of 0.05° (~5.5 km at the equator) using bilinear interpolation prior to modelling analyses. As all predictors shared the same spatial resolution and geographic extent, no additional resampling procedures were required prior to analysis.

To minimize multicollinearity among predictors and improve model interpretability, a Variance Inflation Factor (VIF) analysis was conducted using the vifstep procedure with a threshold value of 10 [23]. The procedure was applied iteratively, whereby the predictor with the highest VIF value was sequentially removed and VIF values were recalculated after each exclusion until all remaining variables exhibited VIF values below the specified threshold. Of the 21 environmental variables initially considered, 12 predictors were retained for subsequent modelling analyses, while 8 variables (air_temp_surf, thetao_depthmax, thetao_depthmean, thetao_depthmin, po4_depthmean, so_depthmean, so_depthmin, and phyc_depthmean) were excluded due to multicollinearity. The retained variables and their corresponding VIF values are presented in Table 1.

Baseline environmental layers represented the period 2000–2020, whereas future environmental conditions were derived from CMIP6 climate projections under the SSP245 scenario for the period 2020–2100 [9,22]. For variables available as annual time-series layers, multi-year mean values were calculated to generate representative baseline and future environmental datasets. This approach is widely used in species distribution modelling studies to characterize long-term environmental conditions and assess potential distributional responses to climate change [5,22].

### 2.3. Species Distribution Modelling

A total of 12 species distribution modelling algorithms were evaluated to identify the most suitable approach for predicting the habitat suitability of *R. undulata*. The tested algorithms included Random Forest (RF), Maximum Likelihood Estimation (MaxLike), Multi-Layer Perceptron Neural Networks (MLP), Classification and Regression Trees (CART), BIOCLIM, Flexible Discriminant Analysis (FDA), Generalized Additive Models (GAM), Generalized Linear Models (GLM), Recursive Partitioning and Regression Trees (RPART), Support Vector Machines (SVM), Boosted Regression Trees (BRT), and Maximum Entropy (MaxEnt).

These algorithms represent a range of machine-learning, regression-based, classification, and environmental envelope methods commonly used in species distribution modelling [5,24]. Model calibration was performed using a bootstrap resampling procedure with three replicate runs. For each replicate, occurrence records were randomly partitioned into training (70%) and testing (30%) datasets to improve model robustness and reduce partitioning bias [25]. In addition, 10,000 randomly generated background points were created within the study area and used consistently across all model runs, providing the background/pseudo-absence information required for algorithms that cannot be fitted using presence-only data. Model performance was evaluated using the Area Under the Receiver Operating Characteristic Curve (AUC) and the True Skill Statistic (TSS) [26].

Because only presence records were available for *R. undulata*, a set of 10,000 randomly generated background points was created across the study area using the gRandom procedure implemented in the sdm package (version 1.2-59). These background points were used consistently across all modelling algorithms and provided the pseudo-absence information required for algorithms that cannot be calibrated using presence-only data.

### 2.4. Habitat Change Analysis

To quantify potential distributional shifts under future climate conditions, habitat suitability maps generated for current and future scenarios were compared using a habitat change analysis [24]. Habitat suitability predictions were converted into binary suitable/unsuitable maps using a threshold value of 0.0688 derived from the maximum True Skill Statistic (TSS) obtained during model evaluation. The same threshold was subsequently applied to both current and future habitat suitability maps to ensure consistency in habitat change assessments. Habitat suitability was then classified into four categories: (i) stable habitat, representing areas suitable under both current and future conditions; (ii) habitat gain, representing newly suitable areas; (iii) habitat loss, representing areas predicted to become unsuitable; and (iv) no habitat, representing areas unsuitable under both scenarios.

### 2.5. Variable Importance and Response Curves

The relative contribution of each environmental predictor was evaluated to identify the primary drivers of *R. undulata* distribution. Variable importance was quantified based on model contribution values generated by the selected SDM algorithm [27]. To further examine species–environment relationships, response curves were produced for each predictor variable. These curves illustrate the effect of individual environmental gradients on habitat suitability while holding all other variables constant, thereby providing insights into the ecological niche characteristics of the species [27,28].

## 3. Results

### 3.1. Species Occurrence Data

**Figure 1:**
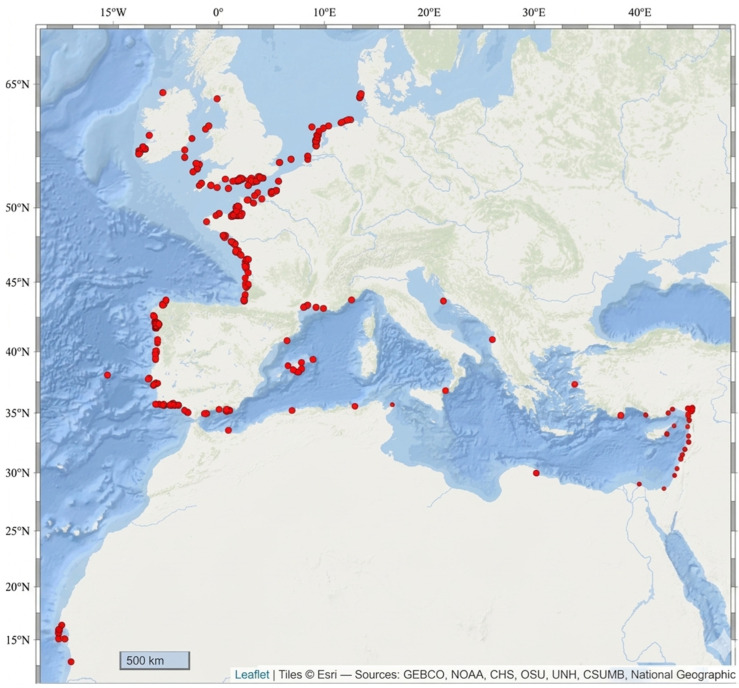
Global and Mediterranean distribution of occurrence records used for modelling the habitat suitability of *R. undulata*. The occurrence records (red dots) on the map were created by the authors using the Leaflet package in R.

Following data validation and filtering, 441 georeferenced occurrence records of *R. undulata* were retained for analysis. The final dataset comprised records from OBIS (184), GBIF (129), iNaturalist (101), iDigBio (5), and published literature (22). Environmental predictor values were successfully extracted for all retained occurrence records, and no additional records were excluded due to missing environmental data. The spatial distribution of occurrence records at both global and Mediterranean scales is shown in Figure 1.

### 3.2. ROC Curve Analysis

**Figure 2:**
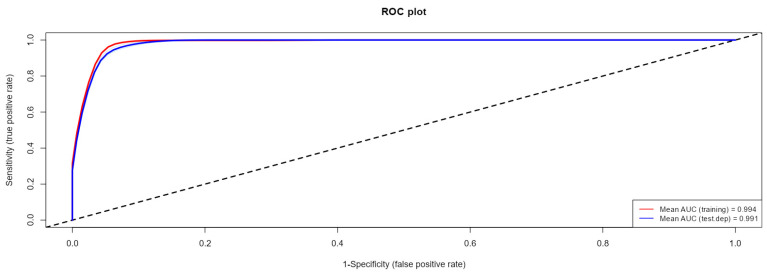
Receiver Operating Characteristic (ROC) curve of the selected Maxent model showing training and testing performance for *Raja undulata*. The dashed diagonal line represents the performance of a random classifier (AUC = 0.5), serving as the reference for no-discrimination.

ROC analysis indicated excellent model performance, with both training and testing AUC values reaching 0.99 (Figure 2). The close agreement between training and testing datasets suggests minimal overfitting and a strong ability of the model to generalize to independent data. These results, together with the high TSS value, confirm the robustness and predictive reliability of the Maxent model for assessing the habitat suitability of *R. undulata*.

### 3.3. Environmental Predictors

To mitigate multicollinearity and avoid model overfitting, a Variance Inflation Factor (VIF) analysis was performed as a preliminary step for variable selection. Predictors displaying high collinearity (VIF ≥ 10) were systematically excluded, resulting in the retention of 13 robust environmental variables for the final habitat suitability models (Table 1).

These retained predictors capture critical oceanographic, physicochemical, and climatic gradients, spanning temperature, salinity, dissolved oxygen, nutrient availability, primary productivity, seawater density, and pH. The selected environmental layers were utilized to simulate both the contemporary and future potential distributions of *R. undulata*. Baseline environmental layers were constructed to represent contemporary conditions based on average values calculated across the 2000–2020 period. To assess the impacts of climate change, future distribution projections (2020–2100) were derived from CMIP6 climate models under the shared socioeconomic pathway SSP2-4.5, representing a medium-emission stabilization scenario. For variables formatted as annual time series, temporal means were calculated across the respective periods to generate representative climatological layers.

### 3.4. Model Performance and Selection

A total of 12 species distribution modelling algorithms were evaluated using AUC and TSS performance metrics. Among the tested models, Maxent achieved the highest predictive performance (AUC = 0.99, TSS = 0.95) and was therefore selected for current and future habitat suitability projections.

An AUC value of 0.99 indicates excellent discriminatory ability between suitable and unsuitable habitats, while a TSS value of 0.95 demonstrates a high level of agreement between observed and predicted distributions. These results indicate that the selected model successfully captured the environmental niche of *R. undulata* and provided highly reliable habitat suitability predictions.

### 3.5. Current Distribution

**Figure 3:**
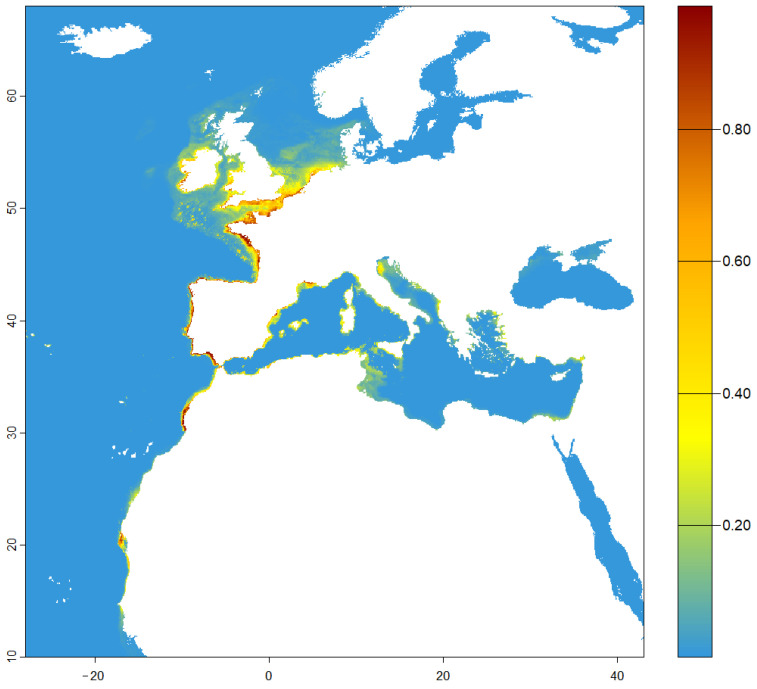
Predicted current habitat suitability of *R. undulata* derived from the Maxent model. Warmer colours indicate higher habitat suitability.

Current distribution modelling revealed that habitat suitability for *R. undulata* is highly heterogeneous across the study area (Figure 3). Areas exhibiting the highest predicted habitat suitability values were concentrated along the Iberian Peninsula, the Bay of Biscay, and the English Channel. Additional areas with relatively high suitability were identified along the Atlantic coasts of Mauritania and Morocco. Within the Mediterranean Sea, suitable habitats were primarily located in the western basin, including the Alboran Sea and waters surrounding the Italian Peninsula. In contrast, the eastern Mediterranean and large portions of the open Atlantic Ocean exhibited comparatively low suitability values. This spatial pattern is consistent with the species’ preference for coastal and continental shelf environments.

### 3.6. Future Distribution

**Figure 4:**
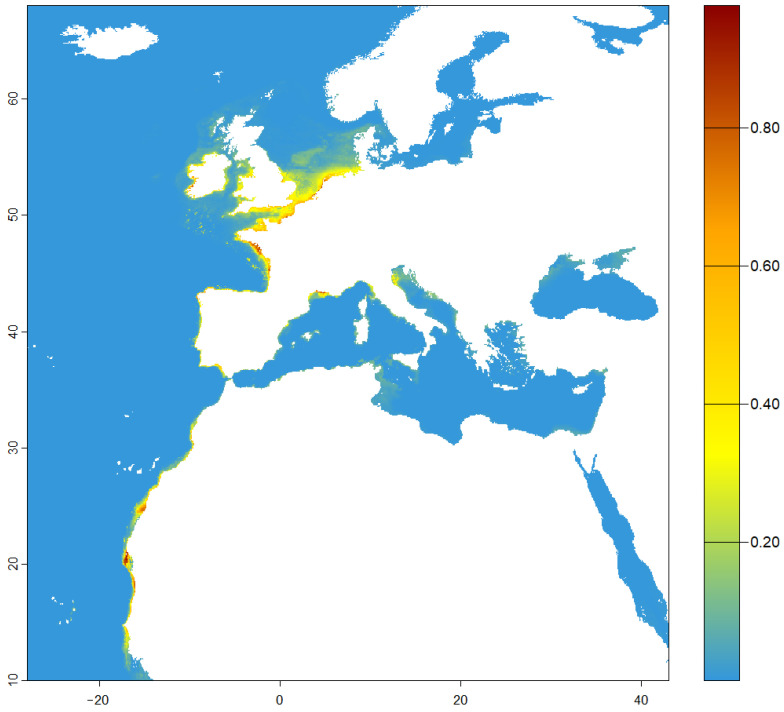
Predicted future habitat suitability of *R. undulata* under the SSP245 climate scenario. Warmer colours indicate higher habitat suitability.

The future distribution model projects a significant contraction of suitable habitats under projected climate change scenarios (Figure 4). Areas of high suitability currently observed in the Bay of Biscay, the English Channel, and the coastal waters of the United Kingdom and Ireland are projected to become largely unsuitable. Similarly, suitable habitats within the western Mediterranean, including the Alboran Sea and surrounding waters, are substantially reduced in future projections.

Suitable habitats are predicted to persist only in a few localized areas, most notably along the coasts of Mauritania and Morocco. This pronounced reduction in habitat suitability highlights the vulnerability of *R. undulata* to future environmental change and suggests that the species may face considerable challenges in maintaining its current distribution range.

### 3.7. Habitat Change Analysis

**Figure 5:**
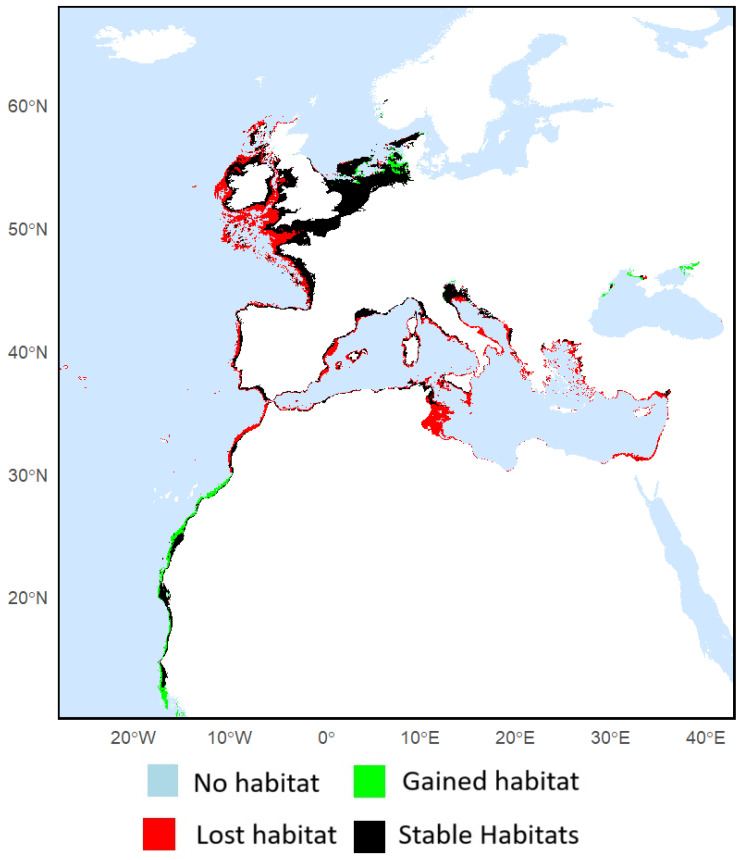
Habitat change analysis showing projected gains, losses, stable habitats, and unsuitable areas for *R. undulata* between current and future climate conditions.

To assess habitat shifts between current and future conditions, a habitat change analysis was conducted using four habitat categories that were visualized using distinct colour codes on habitat change maps (Figure 5). Among areas classified as suitable under either current or future environmental conditions, habitat loss accounted for 57.3%, whereas stable habitats represented 40.5%. In contrast, habitat gain was limited to only 2.2% of the suitable area. These results indicate that projected habitat losses substantially exceed habitat gains, suggesting a marked contraction of suitable habitat for *R. undulata* under future climate conditions.

Habitat loss (red) was the dominant pattern across much of the current distribution, including the Iberian Peninsula, the United Kingdom, and large portions of the western Mediterranean. Stable habitats were limited to scattered areas, indicating that even present-day core habitats may be vulnerable to future environmental change. Areas of habitat gain (green) were highly localized, with the most prominent gains occurring along the coasts of Morocco and Mauritania. This pattern, combined with extensive habitat losses in the northern part of the species’ range, indicates a substantial equatorward (southward) shift in the projected distribution of *R. undulata*.

### 3.8. Variable Contribution and Importance

**Figure 6:**
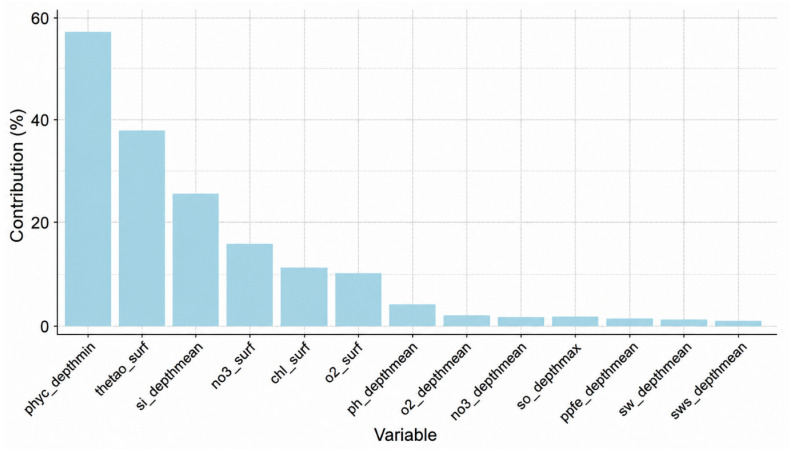
Relative importance of environmental predictors in the Maxent species distribution model for *R. undulata*.

The analysis of variable contributions identified the primary environmental drivers shaping the distribution of *R. undulata* (Figure 6). Variable importance was evaluated using the relative percentage contribution of each predictor to the MaxEnt model. The most influential predictor was phyc_depthmin (minimum phytoplankton concentration), contributing approximately 60% of the total model contribution. The second and third most influential variables were thetao_surf (sea surface temperature), contributing approximately 38%, and si_depthmean (mean silicate concentration), contributing approximately 25%. Additional variables, including no3_surf and chl_surf, also contributed to model performance but with comparatively lower relative contributions.

These results indicate that habitat suitability is primarily associated with productivity-related environmental conditions and sea surface temperature, while other chemical and physical variables play secondary roles in defining the species’ ecological niche.

**Figure 7:**
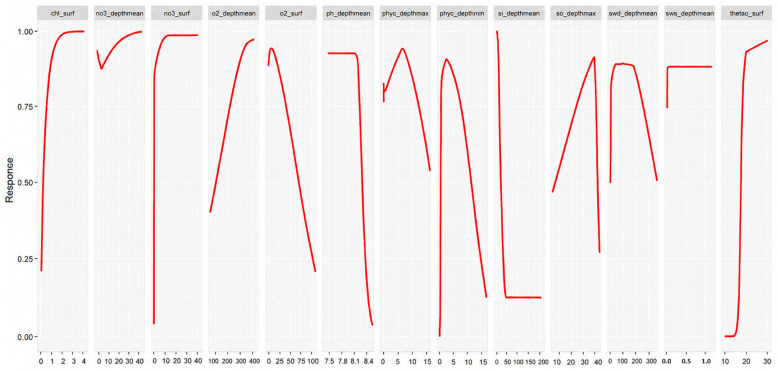
Response curves illustrating the relationships between environmental predictors and predicted habitat suitability of *R. undulata*.

Response curves provided further insight into the environmental preferences of *R. undulata* (Figure 7). The species showed a strong preference for low values of phyc_depthmin, with habitat suitability declining rapidly as phytoplankton concentrations increased. Habitat suitability increased with sea surface temperature and silicate concentration, whereas elevated chlorophyll-a and nitrate concentrations were generally associated with lower suitability. These response patterns indicate that *R. undulata* occupies a relatively narrow environmental niche characterized by specific productivity and oceanographic conditions.

## 4. Discussion

### 4.1. Model Accuracy

The Maxent model employed in this study demonstrated exceptionally high predictive accuracy, as evidenced by AUC values of 0.99 for both training and testing datasets and a TSS of 0.95. The ROC analysis further confirmed the model’s ability to simultaneously achieve high sensitivity and low false-positive rates, while the close agreement between training and testing results indicated minimal risk of overfitting. This performance not only validates Maxent as the most suitable approach among twelve tested algorithms but also confirms its ability to robustly capture the ecological niche of *R. undulata*. Similar findings have been reported for other marine species distribution studies, where Maxent has consistently outperformed alternative algorithms in handling presence-only data and complex ecological interactions [5,28,29]. Nevertheless, potential biases remain, particularly those stemming from the uneven spatial distribution of occurrence records. Incorporating future sampling efforts and fine-scale habitat descriptors such as benthic substrate type could further strengthen predictive reliability [30].

### 4.2. Analysis of the Current Distribution

Current distribution models revealed that *R. undulata* habitats are highly fragmented and concentrated in specific coastal and continental shelf regions. Areas exhibiting the highest predicted habitat suitability values were primarily identified along the Iberian Peninsula, the Bay of Biscay, and the English Channel, as well as in coastal zones of Mauritania and Morocco. In the Mediterranean, the western basin (Alboran Sea and waters surrounding Italy) emerged as a core region, whereas the eastern Mediterranean showed very low suitability. These findings align with the species’ demersal life history and preference for shallow, coastal environments over open-ocean habitats [13,14,15]. Many of these coastal regions are also reported in the literature as being subject to substantial fishing pressure and other anthropogenic disturbances, which may further increase the vulnerability of local populations [11]. This observation highlights the importance of integrating fisheries management with habitat protection in order to mitigate cumulative anthropogenic pressures [31,32].

### 4.3. Future Projections and Habitat Shift

Climate change projections under the SSP245 scenario revealed a dramatic contraction of suitable habitats. Traditional hotspots, such as the Bay of Biscay, the English Channel, and much of the western Mediterranean, are projected to lose suitability almost entirely. Stable habitats were limited to a few scattered patches, while gains were restricted to narrow coastal strips off Mauritania and Morocco. This redistribution indicates a pronounced equatorward (southward) shift in the species’ range. Such a strong contraction of suitable habitats highlights the species’ high sensitivity to future environmental change and suggests severe challenges for its long-term persistence.

Although climate warming is widely associated with poleward range shifts in marine organisms, responses are often species-specific and may be driven by multiple interacting environmental factors rather than temperature alone [33,34]. In the present study, the projected southward redistribution should therefore not be interpreted solely as a direct response to increasing sea surface temperature. Instead, it likely reflects changes in the overall environmental niche of *R. undulata*, including future alterations in productivity-related variables, nutrient regimes, benthic habitat conditions, and prey–resource availability. Because *R. undulata* is a demersal species associated with continental shelf habitats, climate-driven changes in benthic productivity and the distribution of benthic prey communities may indirectly influence habitat suitability. Similar non-poleward distributional responses have been documented in marine species whose habitat suitability is constrained by complex interactions among thermal, trophic, and oceanographic conditions [3,33]. These findings suggest that future distributional shifts may be driven by ecosystem-level changes affecting habitat quality and trophic dynamics rather than by temperature alone, highlighting the importance of incorporating multiple environmental drivers into conservation and fisheries management planning.

### 4.4. Key Drivers and Ecological Implications

Environmental variable analysis identified minimum phytoplankton concentration (phyc_depthmin) as the dominant predictor, contributing nearly 60% to the model’s performance. Sea surface temperature (thetao_surf) and mean silicate concentration (si_depthmean) also played substantial roles, together shaping much of the species’ niche. Response curves revealed that *R. undulata* was associated with relatively low phytoplankton and nutrient concentrations, alongside warmer waters above 20 °C. Given the demersal ecology of the species, the strong influence of phyc_depthmin is unlikely to reflect a direct response to phytoplankton availability. Instead, this variable may act as a proxy for broader environmental conditions, including water-mass characteristics, benthic habitat quality, and prey–resource dynamics that influence the distribution of demersal batoids [13,14]. Areas characterized by lower background productivity may also be associated with specific sedimentary environments and benthic communities preferred by *R. undulata*. Similarly, the importance of silicate concentration may reflect broader nutrient and productivity regimes rather than a direct physiological response. These findings suggest that habitat suitability is influenced by a complex interaction between thermal conditions, productivity-related processes, and benthic ecosystem characteristics, highlighting the species’ sensitivity to environmental changes that alter trophic dynamics and habitat structure [3,16].

The dominance of phytoplankton-related variables over temperature is particularly noteworthy. While sea surface temperature is frequently identified as the primary driver of marine range shifts, the results suggest that habitat suitability for *R. undulata* is more strongly associated with environmental conditions linked to productivity and nutrient availability. It is therefore possible that phyc_depthmin acts not only as a direct ecological driver but also as a proxy for broader oceanographic conditions that characterize suitable habitats. Similar interpretations have been proposed in species distribution modelling studies, where variables with high statistical importance may represent complex environmental gradients rather than direct biological mechanisms [5,29].

The ecological implications of these findings are significant. Unlike many marine species that exhibit poleward shifts in response to warming, *R. undulata* appears to undergo an equatorward redistribution. This atypical response likely reflects the combined influence of productivity, nutrient availability, and temperature rather than thermal preferences alone. Consequently, future distributional changes may not follow the generalized expectation of northward expansion commonly reported for marine fishes [33,34]. Because *R. undulata* is a demersal predator that feeds primarily on benthic invertebrates and small demersal fishes, climate-driven changes in benthic productivity and prey distributions may represent important ecological mechanisms underlying the projected habitat shifts [14]. Alterations in nutrient and productivity regimes could affect the abundance and spatial distribution of benthic prey communities, potentially modifying trophic interactions and habitat quality across continental shelf ecosystems. Such changes may ultimately restructure local food webs, alter competitive dynamics, and reduce ecosystem resilience in both Atlantic and Mediterranean systems [3,16]. Given the species’ vulnerable conservation status, proactive measures such as region-specific fisheries closures, climate-informed marine protected areas, and long-term monitoring programs designed to track habitat suitability shifts are essential to prevent further declines [11,15].

### 4.5. Limitations

A limitation of the present study is the use of long-term averaged environmental variables, which may not fully capture fine-scale temporal variability in oceanographic conditions. While such multi-year averages are widely employed in species distribution modelling to assess broad-scale habitat suitability and climate-driven distributional changes [5,22,35], future studies incorporating seasonal or monthly environmental datasets could provide additional insights into short-term habitat dynamics and temporal variability in habitat use by *R. undulata*. Furthermore, the absence of fine-scale predictors such as benthic substrate characteristics, fishing pressure, and habitat disturbance may limit the representation of local habitat preferences and anthropogenic impacts on *R. undulata* distribution [24,36,37]. Although the model demonstrated excellent predictive performance, the potential effects of spatial autocorrelation and uneven sampling effort cannot be completely excluded. Occurrence records were compiled from multiple biodiversity databases and subjected to extensive quality-control procedures; however, explicit spatial filtering and spatially structured cross-validation approaches were beyond the scope of the present study. Future research incorporating spatially explicit validation frameworks may provide additional insights into the robustness of model predictions and help further reduce potential sampling biases. In addition, future projections were based solely on the SSP245 climate scenario. Although this intermediate pathway provides a realistic framework for assessing potential climate-driven distributional changes, evaluating multiple emission scenarios (e.g., SSP126 and SSP585) would allow a more comprehensive assessment of uncertainty associated with alternative future climate trajectories.

### 4.6. Conservation and Management Implications

From a conservation perspective, the spatial distribution of stable and declining habitats provides valuable guidance for future management actions. Regions such as the Bay of Biscay, the English Channel, and parts of the northeastern Atlantic continental shelf, which are predicted to remain suitable under both current and future conditions, may represent important climate refugia for *R. undulata* and should be considered priority areas for long-term monitoring and conservation. In contrast, areas projected to experience substantial habitat loss, particularly portions of the western Mediterranean and adjacent Atlantic shelf regions, may require enhanced monitoring and adaptive fisheries management measures. The limited habitat gains predicted along parts of the Moroccan and Mauritanian coasts further highlight the importance of international cooperation and climate-adaptive conservation planning capable of accommodating future distributional shifts.

## 5. Conclusions

This study provides the first comprehensive assessment of the current and future habitat suitability of the threatened undulate ray *Raja undulata* across its Atlantic and Mediterranean distribution range under climate change conditions. Among the twelve species distribution modelling algorithms evaluated, MaxEnt achieved the highest predictive performance and identified productivity-related variables, particularly minimum phytoplankton concentration, together with sea surface temperature, as the principal drivers of habitat suitability. Future projections under the SSP245 scenario indicate a substantial reorganization of suitable habitats, with habitat losses (57.3%) greatly exceeding habitat gains (2.2%) and resulting in a net contraction of the species’ suitable range. The projected southward redistribution highlights that future range dynamics are likely to be driven by complex interactions among productivity, nutrient availability, and thermal conditions rather than temperature alone. Areas predicted to remain suitable under both current and future conditions may serve as important climate refugia and should be prioritized for long-term monitoring and conservation. Overall, these findings emphasize the need to incorporate climate-driven habitat shifts into fisheries management, conservation planning, and adaptive monitoring strategies to support the long-term persistence of *R. undulata* in a rapidly changing marine environment.

## Figures and Tables

**Table 1:** Environmental predictors retained after VIF analysis and used for species distribution modelling of *R. undulata*.

Ecological Category	Variable Code	Description	Ecological Relevance	VIF
Primary Productivity	phyc_depthmin	Minimum phytoplankton concentration (mmol m^−3^)	Reflects minimum phytoplankton availability and low-productivity conditions; identified as the most influential predictor of *R. undulata* habitat suitability.	6.23
Sea Surface Temperature	thetao_surf	Sea surface temperature (°C)	Influences physiological performance, metabolic processes, and species distribution; a major driver of habitat suitability.	4.45
Nutrient Availability	si_depthmean	Mean silicate concentration (mmol m^−3^)	Indicates nutrient availability and primary production dynamics, particularly associated with diatom productivity.	3.08
Nutrient Availability	no3_surf	Surface nitrate concentration (mmol m^−3^)	Reflects nutrient conditions in surface waters and local primary productivity.	4.63
Primary Productivity	chl_surf	Surface chlorophyll-a concentration (mg m^−3^)	Proxy for phytoplankton biomass and ecosystem productivity.	3.27
Dissolved Oxygen	o2_surf	Surface dissolved oxygen concentration (mmol m^−3^)	Reflects oxygen conditions in surface waters that may affect species occurrence.	3.03
Ocean Acidification	ph_depthmean	Mean pH	Represents seawater acidity/alkalinity, influencing physiological performance and ecological tolerance.	3.30
Dissolved Oxygen	o2_depthmean	Mean dissolved oxygen concentration (mmol m^−3^)	Indicates average oxygen availability and habitat quality for marine organisms.	3.22
Nutrient Availability	no3_deptmean	Mean nitrate concentration (mmol m^−3^)	Represents background nutrient conditions that may influence productivity and trophic structure.	2.18
Salinity Regime	so_depthmax	Maximum salinity (psu)	Represents the upper salinity thresholds in the water column, influencing osmoregulatory capacity and spatial distribution limits.	5.75
Primary Productivity	phyc_depthmax	Maximum phytoplankton concentration	Indicates peak productivity conditions and phytoplankton biomass availability.	5.68
Seawater Physics	swd_depthmean	Mean seawater density (kg m^−3^)	Influences water-column structure, mixing processes, and habitat characteristics.	1.02
Salinity Regime	sws_depthmean	Mean sea surface salinity (psu)	Reflects salinity regime affecting osmoregulation and species distribution.	1.14

## Data Availability

The data can be given upon responsible request.
